# Revolutionizing Glucose Monitoring: Enzyme-Free 2D-MoS_2_ Nanostructures for Ultra-Sensitive Glucose Sensors with Real-Time Health-Monitoring Capabilities

**DOI:** 10.1021/acsomega.3c10117

**Published:** 2024-04-25

**Authors:** Mustri Bano, Gowhar A. Naikoo, Fatima BaOmar, Jahangir Ahmad Rather, Israr U. Hassan, Rayees Ahmad Sheikh, Palanisamy Kannan, Murtaza M. Tambuwala

**Affiliations:** † Department of Mathematics and Sciences, College of Arts and Applied Sciences, Dhofar University, Salalah, PC 211, Oman; ‡ Department of Chemistry, Sri Pratap College Srinagr-190001 Kashmir, India; § Department of Chemistry, AAAM Degree College Bemina Srinagar − 190018 Kashmir, India; ∥ College of Biological, Chemical Sciences and Engineering, Jiaxing University, Jiaxing 314001, PR China; ⊥ Lincoln Medical School, University of Lincoln, Brayford Pool Campus, LN6 7TS Lincoln, U.K.; △ College of Pharmacy, Ras Al Khaimah Medical and Health Sciences University, Ras Al Khaimah, United Arab Emirates

## Abstract

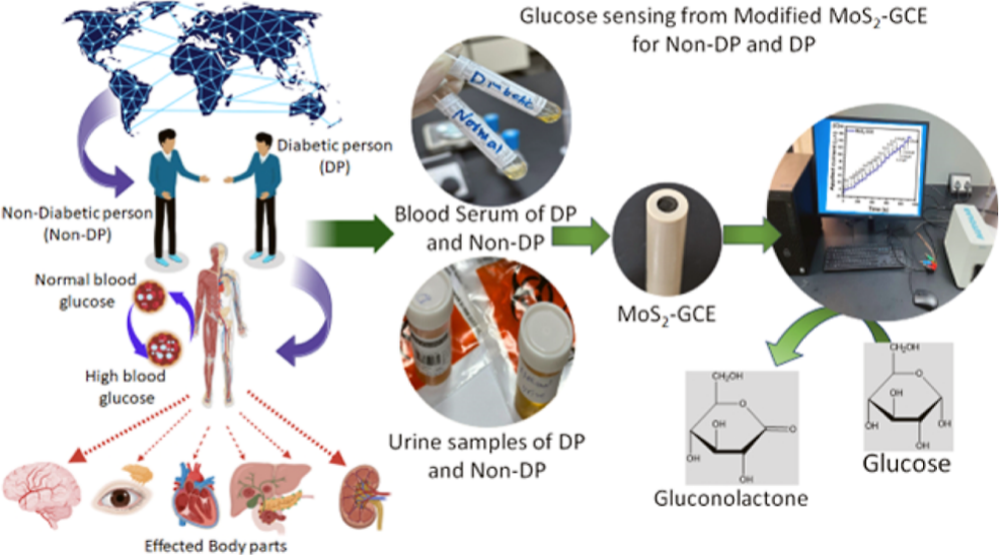

The growing requirement for real-time monitoring of health factors such as heart rate, temperature, and blood glucose levels has resulted in an increase in demand for electrochemical sensors. This study focuses on enzyme-free glucose sensors based on 2D-MoS_2_ nanostructures explored by simple hydrothermal route. The 2D-MoS_2_ nanostructures were characterized by powder X-ray diffraction, energy-dispersive X-ray spectroscopy, scanning electron microscopy, transmission electron microscopy, Raman spectroscopy, and XPS techniques and were immobilized at GCE to obtain MoS_2_–GCE interface. The fabricated interface was characterized by electrochemical impedance spectroscopy which shows less charge transfer resistance and demonstrated superior electrocatalytic properties of the modified surface. The sensing interface was applied for the detection of glucose using amperometry. The MoS_2_–GCE-sensing interface responded effectively as a nonenzymatic glucose sensor (NEGS) over a linearity range of 0.01–0.20 μM with a very low detection limit of 22.08 ng mL^–1^. This study demonstrates an easy method for developing a MoS_2_-GCE interface, providing a potential option for the construction of flexible and disposable nonenzymatic glucose sensors (NEGS). Moreover, the fabricated MoS_2_–GCE electrode precisely detected glucose molecules in real blood serum and urine samples of diabetic and nondiabetic persons. These findings suggest that 2D-MoS_2_ nanostructured materials show considerable promise as a possible option for hyperglycemia detection and therapy. Furthermore, the development of NEGS might create new prospects in the glucometer industry.

## 1 Introduction

The World Health Organization estimates that over 350 million people worldwide have diabetes, and diabetes is expected to become the sixth leading cause of death by 2030, based on current projections.^1−3^ Diabetes affects millions of individuals worldwide, particularly those with middle- or low incomes. It can lead to serious problems such as renal failure, eyesight loss, heart attacks, strokes, and the need for limb amputations if not treated properly.^4^ Individuals with diabetes must monitor their blood glucose levels in order to manage illness and lessen the negative impacts of numerous diabetes-related ailments. Numerous investigations are being conducted to produce more precise glucose testing procedures due to the limitations of the present diagnostic methods. The development of sophisticated glucose biosensors is in high demand in society. The primary goal of research has been to improve detecting capabilities and biocompatibility over present technologies, hence opening up new options for more efficient glucose sensors. Nanotechnology has played a critical part in these efforts, harnessing nanomaterials’ unique features to expand sensor surface area and improve electrode catalytic capabilities.

Electrochemical glucose sensors have sparked considerable attention among the many detection methods due to their low cost, real-time detection capabilities, and simple operation. The development of glucose sensors may be divided into four major generations.^5−10^ The technology used in the first generation of sensors entailed immobilizing a catalytic enzyme, such as glucose oxidase (GOx), onto an electrode. First-generation glucose sensors met interference from electroactive compounds in the blood, such as uric acid, ascorbic acid, and a variety of other medicines that may be present in the circulation.^4,11^ To enhance the electron transport mechanism, the second generation of glucose sensor technology proposed using a nonphysiological, artificial mediator to replace the first-generation mediator, oxygen.^4^ Although second-generation sensors have managed to overcome some of the problems of their predecessors, their performance and sensitivity remained reliant on variations in medium pH as well as temperature and humidity on the electrode surface. The third generation of glucose sensor technology aimed to do away with the necessity for a reaction mediator, allowing for direct electron transmission from the enzyme to the electrode. The enzyme was immobilized on the electrode to achieve this.^10^ While the third-generation sensors were expected to improve on their predecessors, they continued to confront issues due to the enzyme’s vulnerability to conditions such as temperature, humidity, and interference. The shortcomings associated with these third-generation enzyme-based sensors for glucose prompted researchers to seek enzyme-free detection. This research resulted in the creation of nonenzymatic glucose (NEG) sensors that enable the direct oxidation of glucose on the electrode surface. The fourth generation of glucose sensor technology is NEG sensors.

Two-dimensional (2D) materials are a diverse group that includes monolayer carbon compounds, chalcogenides, nitrides, phosphides, halides, layered silicate minerals, and oxyhalides.^12,13^ Because of its unique and technologically exciting features, MoS_2_ stands out as an extraordinary material within the wide domain of 2D transition-metal dichalcogenides, with a rich history of investigation and use. MoS_2_ is an isostructural counterpart of graphene with a chemically adaptable lamellar structure that leads to a wide range of uses. It is widely regarded as one of the most promising 2D nanomaterials.^14^ It has a layered structure comparable to graphite with 0.32 nm interlayer spacing. Its bandgap ranges from 1.23 to 1.8 eV, making it helpful in a variety of applications including microelectronics.^15^ Several nano(bio)composite electrodes using MoS_2_ have been developed in recent years to accomplish very sensitive electrochemical detection of diverse redox chemicals linked with food, biomolecules, medicines, environmental contaminants, inorganic ions, and gaseous molecules.^16,17^ The electrochemical experiments show that MoS_2_ nanostructures have significant electrocatalytic activity and a wide range of analytical adaptability in addition to their role as catalysts in hydrogen evolution and oxygen reduction processes.^18−21^ This greatly contributes to the development and enhancement of improved functional electrochemical platforms for sensing applications in a variety of fields, such as food safety, medicines, biochemical analysis, and environmental monitoring. 2D-MoS_2_ nanostructures provide synergistic effects when used as an electrode material, improving conductivity, catalytic activity, and biocompatibility. This acceleration allows for more efficient signal transduction via biorecognition mechanisms, especially with selected signal tags.^22,23^

In this perspective, current work designates the synthesis of 2D-MoS_2_ nanocatalyst using a one-step hydrothermal method and immobilized on GCE for uncovering of glucose sensitivity. The electrocatalytic effectiveness of the designed 2D-MoS_2_-GCE interfaces for real-sample glucose detection was evaluated by using cyclic voltammogram (CV) and amperometry. The proposed method provides a fast and sensitive platform for assessing glucose concentration in real blood serum and urine samples of diabetic and nondiabetic persons while limiting interference from other chemicals. Early glucose detection with modified electrodes allows for timely treatment, avoiding potentially serious health problems.

## 2 Experimental Section

### 2.1 Synthesis of 2D-MoS_2_ Nanostructures

**Scheme 1:**
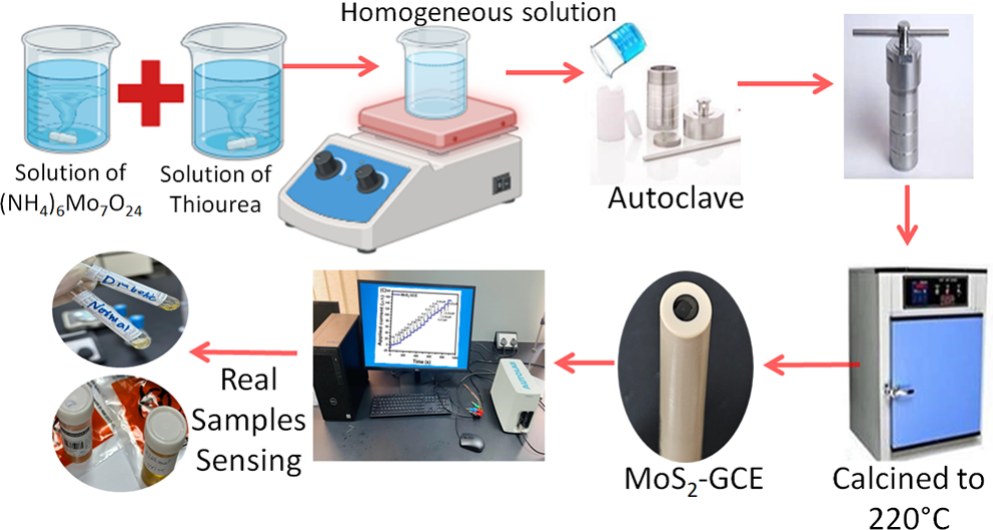
Synthetic Representation of 2D-MoS_2_ Nanostructured Materials “Photograph Courtesy of ‘Mustri Bano’. (Copyright 2024.”)

The 2D-MoS_2_ nanostructures were synthesized by using a one-step hydrothermal method. In a typical method, an aqueous solution containing millimoles of ammonium heptamolybdate tetrahydrate ((NH_4_)_6_Mo_7_O_24_) and millimoles of thiourea were dissolved in 40 mL of ultrapure water under vigorous stirring for half an hour to form homogeneous solution before transferring it to a 50-mL Teflon-lined stainless-steel autoclave and calcined to 220 °C for 24 h in a furnace. The resulting sample was collected and washed with distilled water and ethanol, and dried in an oven before characterization^24,25^ (Scheme 1).

All electrochemical studies were performed at ambient temperature using a three-electrode setup composed of a Pt counter electrode, an Ag/AgCl reference electrode, and a 5.0-mm glassy carbon active/working electrode (GCE). The functioning electrode was created by altering the surface of a bare glassy carbon electrode (b-GCE) with a dispersion of 2D-MoS_2_ nanostructures. This dispersion was made by dissolving 5.0 mg of manufactured 2D-MoS_2_ in 10 mL of ethanol ultrasonically. The GCE surface was polished with alumina slurry before being sonicated in ethanol for 10 min, rinsed with distilled water, and dried at room temperature. The solvent was allowed to evaporate at room temperature after application of the dispersed mixture (10 μL) on the GCE surface. To improve electrode efficiency, the modified 2D-MoS_2_–GCE or MoS_2_–GCE was chemically activated in a 0.1 M nitric acid solution using cyclic potential sweeps that ranged from −1.0 to +2.0 V to obtain stable voltammograms. After each electrochemical experiment, the changed electrode surfaces were washed with a 1.0 mM NaOH solution by sweeping the voltage in the reverse direction (from 1.0 to 0.0 V).

### 2.2 Instrumentations

To analyze the produced 2D-MoS_2_ nanostructures, many analytical approaches were used. Powder X-ray diffraction (P-XRD) was performed using X Pert PRO X-ray diffraction with Cu Kα radiation. A Jobin Yvon LabRam HR spectrometer with an Ar laser at 632 nm was used to acquire Raman spectra from numerous sample sites. XPS experiments were performed with an Omicron spectrometer with Al Ka as the X-ray source (1486.6 eV) to examine the atomic ratio and determine the elemental makeup. A JEOL JSM-6510LA electron microscope was used for morphological research [scanning electron microscopy (SEM) and EDX]. Transmission electron microscopy (TEM) examination using a JEM1400 apparatus was used to conduct detailed research into the structure, morphology of the nanostructured materials, and size distribution. The nanostructured materials were synthesized using a force air drying Oven LBX OVF Series. Autolab PGSTAT204 FRA32 M instrument was used for electroanalytical experiments (CV and amperometry).

## 3 Results and Discussion

### 3.1 X-Ray Diffraction and EDX Analysis of 2D-MoS_2_ Nanostructures

The X-ray diffraction (XRD) patterns obtained from the as-synthesized 2D-MoS_2_ sample are shown in Figure S1A. The distinctive diffraction peaks of 2D-MoS_2_, which appear at roughly 14.0, 33.2, 39.6, and 58.9° angles are matching and recognized with the crystallographic planes (002), (100), (103), and (110) of 2D-MoS_2_. These assignments correspond to the relevant entries in the JCPDS database with the reference code (JCPDS 37-1492).^26^

Energy dispersive X-ray (EDX) spectrum measurement was used to characterize the overall chemical composition of the 2D-MoS_2_ nanostructures, and the result is shown in Figure S1B. Strong peaks associated with S and Mo are found in the spectrum. The quantitative analysis shows that Mo/S is about 1:2, consistent with atomic ratio of MoS_2_ structure, which proves that the synthesized products are 2D-MoS_2_ nanostructures.

### 3.2 Morphological Characterization of Synthesized 2D-MoS_2_ Nanostructures

**Figure 1:**
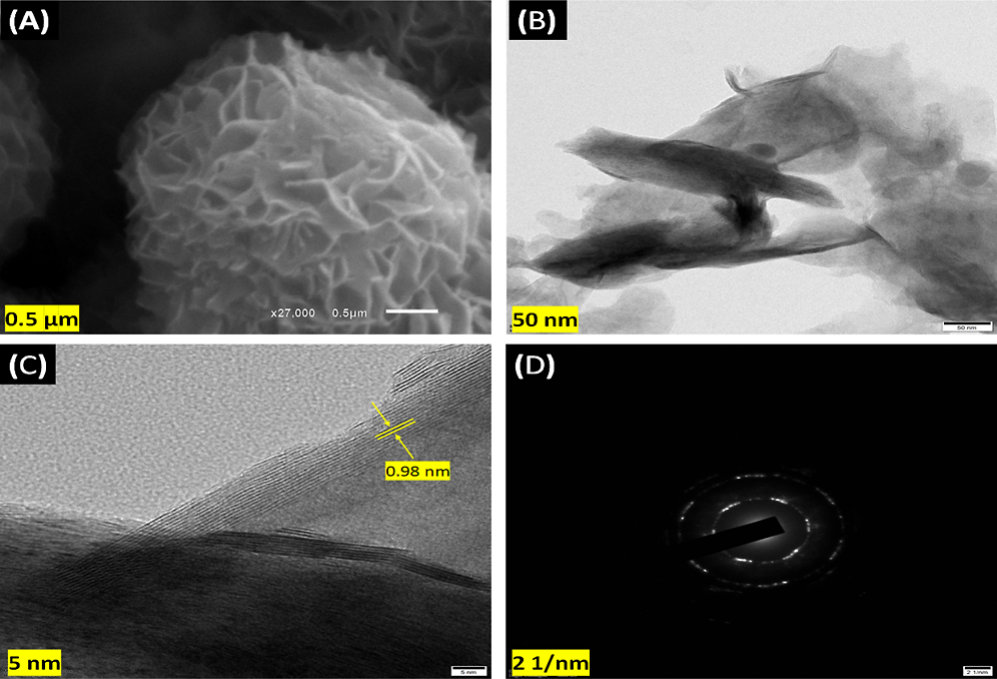
(A) SEM image at 0.5 μm scale. (B) TEM image at 50 nm. (C) HRTEM image at 5 nm of 2D-MoS_2_ nanostructures. (D) SEAD pattern of 2D-MoS_2_ nanostructures.

The surface morphology of the 2D-MoS_2_ nanostructure material is studied in detail using SEM at a scale of 0.5 μm. MoS_2_ has a complex layered structure with noticeable contrasts between the Mo and S atoms. Layers can be seen stacking together to produce a three-dimensional structure with obvious interlayer spaces (Figure 1A).^27^ The HRTEM representation of 2D-MoS_2_ (Figure 1B) reveals a translucent, wrinkled sheet-like structure with a few stacked layers, which is consistent with the SEM results. According to lattice fringe patterns (Figure 1C), the interplanar distance of the MoS_2_ nanosheets was calculated to be 0.72 nm, showing an increase in the typical *d*-spacing (0.98 nm). The intercalation of guest molecules like as ammonia and water between the layers is responsible for the increased interlayer gap.^28^ SAED is a diffraction approach for determining crystal structures and crystal defects in a material. Figure 1 (D) depicts an SAED picture of 2D-MoS_2_ nanosheets in a discrete location, indicating their crystalline character, which coincides with the XRD data.^29^

### 3.3 XPS and Raman Spectroscopic Characterization of 2D-MoS_2_ Nanostructures

The XPS survey spectrum of 2D-MoS_2_ is shown in Figure S2A, with clear peaks corresponding to S and Mo components and no detected impurities. Oxygen (O) in the spectrum might be attributable to molybdate precursors or surface oxidation during XPS experiments. The presence of physically adsorbed oxygen molecules is especially in the annealed sample. The enhanced O 1s atom at ∼532 eV is attributed to these adsorbed oxygen species. This oxygen incorporation increases the intrinsic conductivity of MoS_2_ and, as a result, improves catalytic activity. The Mo 3d XPS spectrum in Figure S2B displays four peaks at 226.5, 227.0, 233.5, and 236.3 eV that correspond to the Mo 3d_5/2_ (1T phase), Mo 3d_5/2_ (2H phase), Mo 3d_3/2_ (1T phase), Mo 3d_3/2_ (2H phase), and Mo^6+^ orbitals, respectively.^30^ The S 2p area has discrete peaks at 161.8, 162.4, and 163.2 eV, which correspond to S^2–^ 2p3/2, S^2–^ 2p_1/2_, and S^2–^ 2p_3/2_ (Figure S2C). When contrasted with pure MoS_2_, these peaks demonstrate a modest chemical shift toward lower binding energy levels. This change significantly shows that an electron charge transfer from sulfur to MoS_2_ occurred. In the corresponding Raman spectrum, two strong peaks at 384 and 410 cm^–1^ are observed,^31^ which are ascribed to the E_2g_ in-layer shifting of Mo and S atoms and A_1g_ modes of vibration for out-of-plane symmetric shifts of S across the *c*-axis, respectively in Figure S2D.

### 3.4 Characterization of 2D-MoS_2_–GCE Sensing Interface

**Figure 2:**
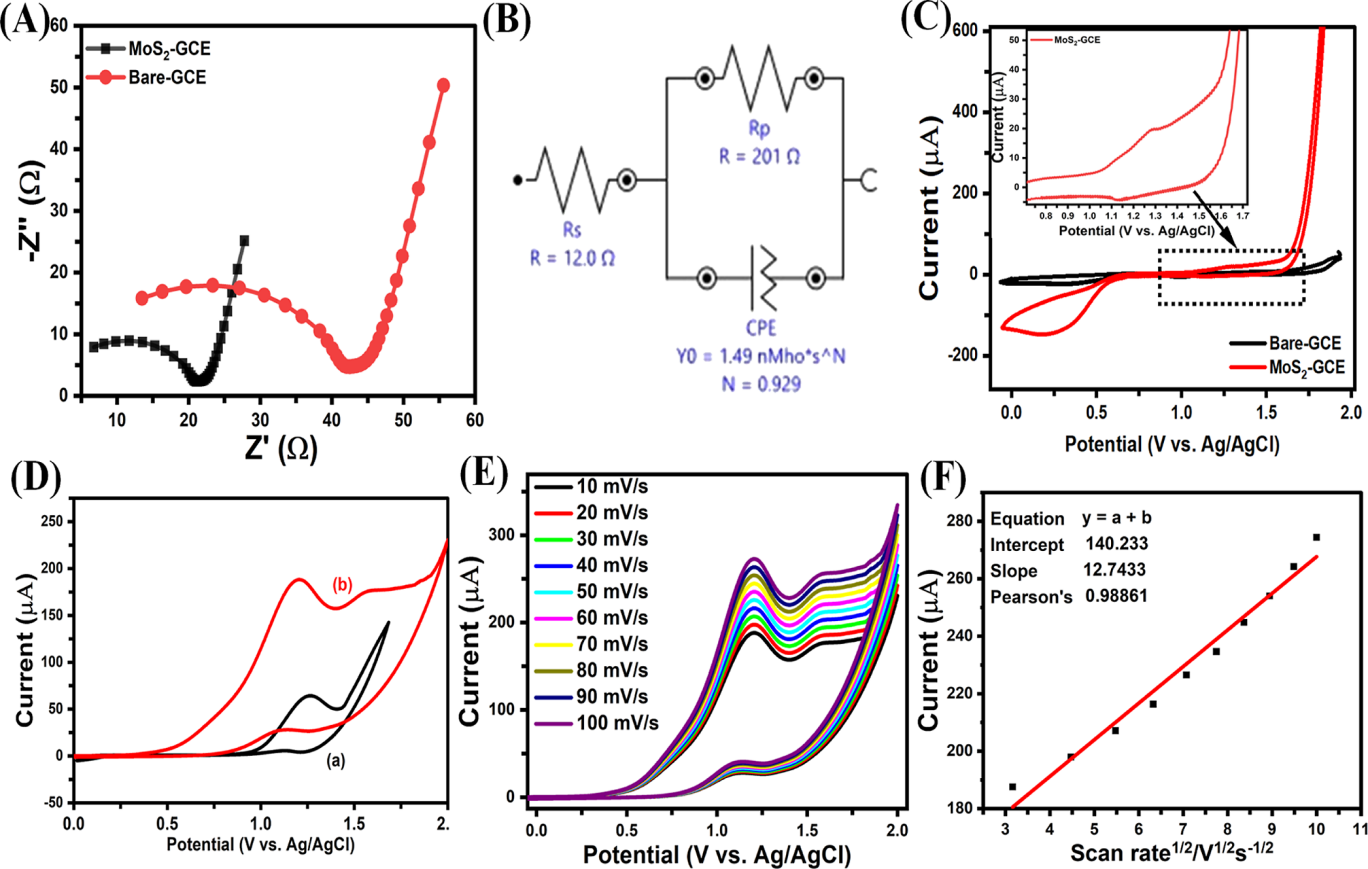
(A) Nyquist plot of GCE and MoS_2_–GCE with the (B) equivalent circuit in 1 mM Fe(CN)_6_^3–/4–^ in 0.1 M KCl solution. Electrochemical oxidation response of (a) bare-GCE, and (b) MoS_2_–GCE electrodes in the absence (C) and the presence of glucose (50 μM) (D) at a potential range of 0.0 to 2.0 V at a scan rate of 10 mV s^–1^. (E) MoS_2_–GCE electrode toward the glucose (50 μM) oxidation at various scan rates (10–100 mV s^–1^) and (F) corresponding linearity plot obtained using glucose oxidation current (μA) versus various scan rates.

The electrochemical characterization of as-fabricated MoS_2_-GCE sensing interface was accessed by electrochemical impedance spectroscopy technique.^28^ The electrochemical interfacial properties of unmodified and modified GCE electrode were studied in 0.1 M KCl solution containing 10 mM [Fe(CN)_6_]^3–/4–^ redox system and the scanning frequency was set from 0.1 Hz to 100 kHz at an amplitude of 5 mV. The obtained results were fitted with Randles–Sevcik circuit, which is shown in Figure 2B. The impedimetric response shows two characteristic parts, linear and a semicircular corresponds to charge transfer resistance (*R*_ct_), and the rate of diffusion-limited electron transfer process, respectively. Moreover, in the high-frequency region, the intercept at the *x*-axis fitting displays as the inset. The bare GCE electrode shows a larger semicircle portion with *R*_ct_ value of 32.61 Ω and indicating the noticeable electron transfer process occurred at the surface (Figure 2A; curve a). Remarkably, it was displayed that there was a clear reduction in faradaic-current response of MoS_2_-GCE modified substrate owing to remarkable reduction in *R*_ct_ value (17.29 Ω), revealed effective immobilization of MoS_2_ at GCE surface (Figure 2A; curve b). After functionalization of GCE by MoS_2_-GCE, the *R*_ct_ value of modified sensing interface there is appearance of rapid heterogeneous faster electron transfer kinetics,^32^ which is mainly attributed to its high surface conductivity and higher charge/discharge capacitance of deposited MoS_2_. The enhanced catalytic performance can be attributed to many causes, including the abundance of edge sites that expose S edge atoms and the significant degree of exfoliation, which results in a decreased number of layers. This exfoliation promotes efficient electron transport between the electron-rich margins and the electrode surface. The electron transfer rate constant *k*_app_ was calculated from the following equation^33^
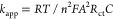
where *n* denotes the “number of electrons involved (*n* = 1),” C represents the “concentration of redox probe,” *A* denotes geometric area (cm^2^) of electrode, *R* represents the “molar gas constant”, *F* represents the “Faraday constant”, and *T* represents the “absolute temperature.” The *k*_app_ value of different electrodes such as GCE and MoS_2_–GCE sensing interface was about 1.2 × 10^–7^ and 4.5 × 10^–7^ s^–1^, respectively, which obviously confirmed that the electron transfer rate of MoS_2_-GCE was about four times higher than GCE interface.

### 3.5 Determination of Glucose at MoS_2_–GCE-Modified Surface

The electrocatalytic activity of the 2D-MoS_2_ nanostructures toward glucose oxidation was studied by the CVs method. As shown in Figure 2C, the bare-GCE and MoS_2_-GCE modified electrode were recorded in the absence of glucose (50 μM) in 0.1 M NaOH. Notably, no oxidation or reduction peak was observed at bare GCE (Figure 2C, black line) depicts that the absence any active sites on the surface of GCE. On the other side, the MoS_2_-GCE was showed an improved redox peak of Mo^2+^/Mo^3+^ in the potential window between 1.0 and 1.5 V in 0.1 M NaOH. The oxidation and reduction peaks of Mo^2+^/Mo^3+^ redox transformation was appeared at 1.26 and 1.12 V, respectively (Figure 2C, red line). The formal potential (E°′ = Ep_a_ + Ep_c_/2) of the Mo^2+^/Mo^3+^ redox peak on the MoS_2_-GCE electrode was found to be +0.07 V which was in agreement with other reports. As shown in Figure 2D, the bare-GCE and MoS_2_-GCE modified electrode were recorded in the presence of glucose (50 μM) in 0.1 M NaOH. As can see the Figure 2D, the bare-GCE electrode displayed a small irreversible oxidation response toward the oxidation of glucose at 1.28 V with a current value of 67.4 μA (Figure 2D, curve a) depicts that the GCE electrode was less efficient. On the other side, the MoS_2_-GCE was showed an onset oxidation potential at 0.5 V and followed by enhanced glucose oxidation response at 1.22 V with the current value of 185.2 V, which 2.74 times higher than the bare GCE electrode (Figure 2D, curve b). Notably, the observed onset oxidation current response was 0.5 V less than that of the bare GCE electrode, indicating the superior performance of 2D-MoS_2_ nanostructures. The observed higher oxidation response on 2D-MoS_2_ nanostructures was mainly attributed to the following reasons; (i) the 2D-MoS_2_ nanosheet structures offer a large specific (active) surface area; (ii) the network-like interconnected 2D-MoS_2_ nanosheet structures were provided good electronic conductivity, which enable the consistent electrical contacts toward the glucose molecules; (iii) their compatibility that is, 2D-MoS_2_ nanosheet structures were possessed large amount of mesopores, makes them as active materials and contributes considerably enhanced electro-oxidation of glucose. Next, in order to evaluate whether the glucose oxidation process on the 2D-MoS_2_ nanosheet structures electrode occurred beneath diffusion or adsorption control, the effects of scan rate on peak current response of glucose were studied in 0.1 M NaOH (supporting electrolyte) containing 50 μM glucose in a 0.0 to 2.0 V potential range at 10–100 mV s^–1^ scan rate (Figure 2E). It was observed that with increase in scan rate, peak currents were increased and peak potential slightly shifted to positive direction confirms the diffusion-controlled oxidation of glucose at fabricated 2D-MoS_2_ nanosheet structures. The corresponding linearity plot was obtained by plotting the glucose oxidation current (μA) versus various scan rates (Figure 2F).

### 3.6 Amperometric Detection of Glucose at MoS_2_–GCE-Modified Interface

**Figure 3:**
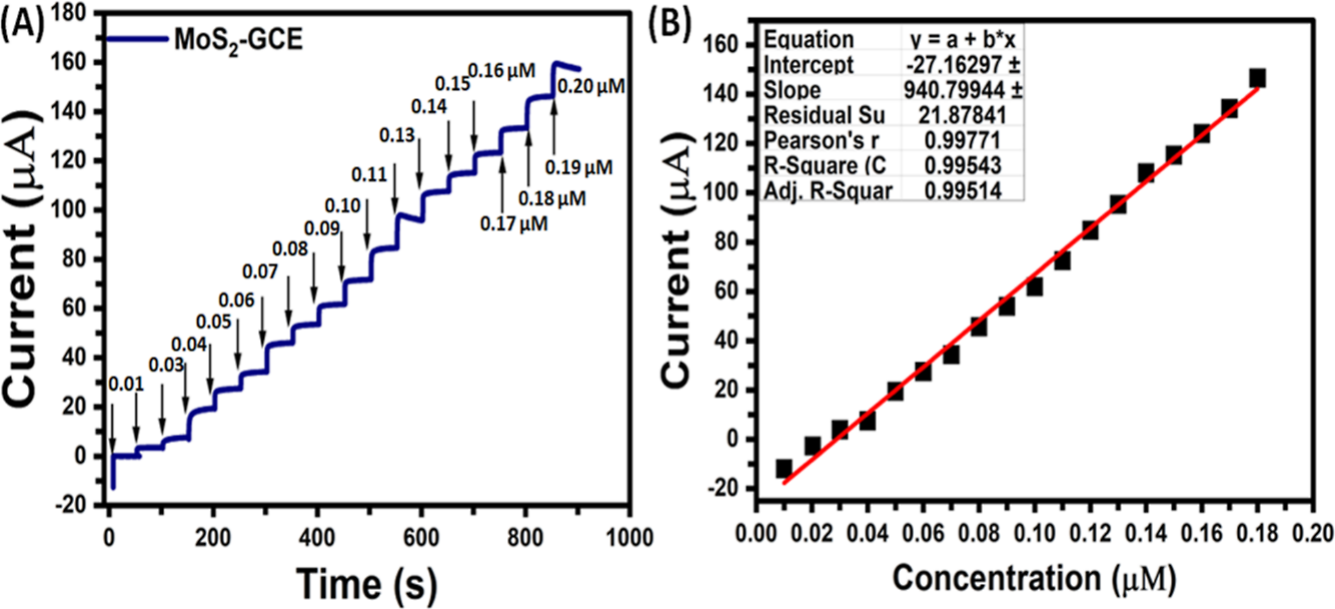
(A) Amperometry *i*–*t* curve and (B) glucose concentrations (sequential addition of glucose from 0.01 to 0.20 μM) versus current response plot with 0.1 M NaOH solution.

The sensitivity of MoS_2_–GCE nanostructures composite electrode was studied toward the amperometric detection of glucose using steady-state conditions. Figure 3 illustrates the amperometric (*i*–*t*) curve responses obtained toward the oxidation of glucose at MoS_2_–GCE nanostructures electrode in 0.1 M NaOH solution with constant stirring mode at an applied potential of −0.60 V. As can be seen in Figure 3A, the early steady-state amperometric *i*–*t* curve response was mainly attributed to the addition of 0.01 μM glucose, and thereafter the successive addition of 0.01 μM glucose upon the further steps with a sample interval of 50 s; the current responses were linearly enhanced and a steady state current response was acquired within 3 s which demonstrated a rapid electro-oxidation process of glucose at this electrode. We estimated that ∼6.1 μA current responses were obtained for single addition of 0.01 μM glucose at the MoS_2_-GCE nanostructure composite electrode.

The observed highly stable amperometric *i*–*t* current response with higher sensitivity of glucose at the MoS_2_-GCE nanostructures composite modified electrode showed that this electrode can be effectively used for the sensitive detection of 0.01 μM glucose. The amperometric *i*–*t* current responses were increased linearly with respect to glucose concentrations from 0.01 to 0.20 μM (Figure 3A), and the detection limit (LOD) was derived from the standard deviation of the baseline current, which is about 2.2 × 10^–5^ μM (S/N = 3). The sensitivity of MoS_2_-GCE nanostructures composite modified electrode was found to be 610.0 μA/μM. The linear plot obtained for amperometric current responses versus various concentrations of glucose was shown in Figure 3B. A good linear curve was obtained with a correlation coefficient of 0.999. It is important to mention here that a noticeable LOD (2.2 × 10^–5^ μM) was attained by using a non-noble MoS_2_-GCE nanostructures composite electrode.

**Figure 4:**
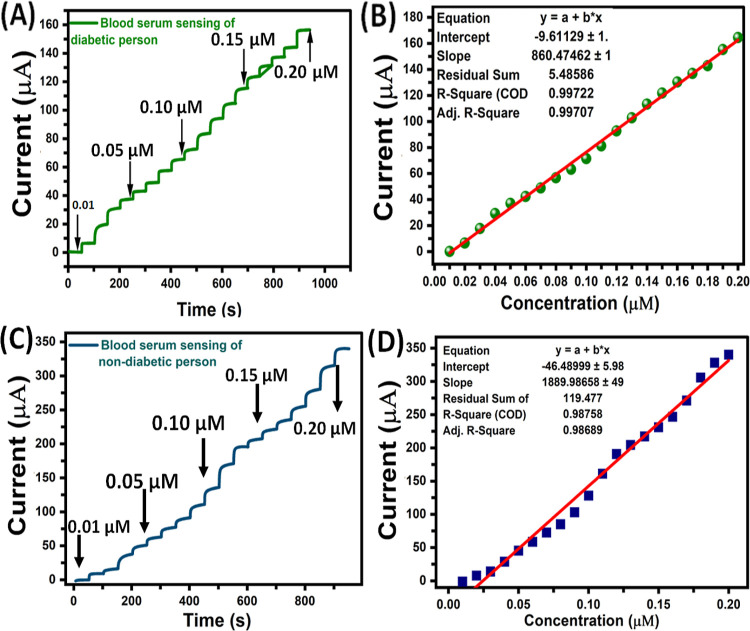
(A) Amperometry spectra and (B) glucose concentration vs current response plot present in blood serum response of diabetic person with 0.1 M NaOH solution. (C) Amperometry spectra and (D) glucose concentration versus current response plot present in blood serum response of nondiabetic person with 0.1 M NaOH solution.

On the other side, we carried out a similar analysis using a diabetic patient’s blood serum sample; we obtained fairly similar steady state amperometric *i*–*t* current responses for all the glucose additions (Figure 4A) from 0.01 to 0.2 μM and the corresponding linear plot obtained for amperometric current responses vs various concentrations of glucose was shown in Figure 4B. A good linearity was obtained with a correlation coefficient of 0.997. The injections of various concentrations of glucose in blood serum sample from the diabetic patient’s and corresponding recovery results were summarized in Table 1.

**Table 1:** Detection of Glucose in Blood Serum Samples Collected from a Diabetic Patient

added (mM)	found concentration (mM)	mean recovery (%)	RSD (%, *n* = 3)
0.01	0.00026	94.50	0.000723
0.05	0.039737	99.97	0.003441
0.1	0.089737	99.98	0.006732
0.15	0.139737	99.99	0.001621
0.2	0.189737	99.99	0.000948

For comparison, the detection of glucose was also performed in blood serum samples that were collected from nondiabetic person using MoS_2_–GCE interface by amperometry (Figure 4C). We have added the similar concentrations of glucose (0.01 to 0.2 μM) into the blood serum samples of nondiabetic person’s sample; the obtained amperometric *i*–*t* curve responses were fairly less sensitive, and the corresponding linearity was distorted with the *R*^2^ value of 0.987 (Figure 4D), which obviously indicate the clear difference during the detection of glucose. The observed results indicate that the MoS_2_–GCE sensing interface showed a glucose detection limit of 1.83 μA/μM in nondiabetic patients serum samples compared to diabetic patient’s serum samples (1.07 μA/μM).

**Figure 5:**
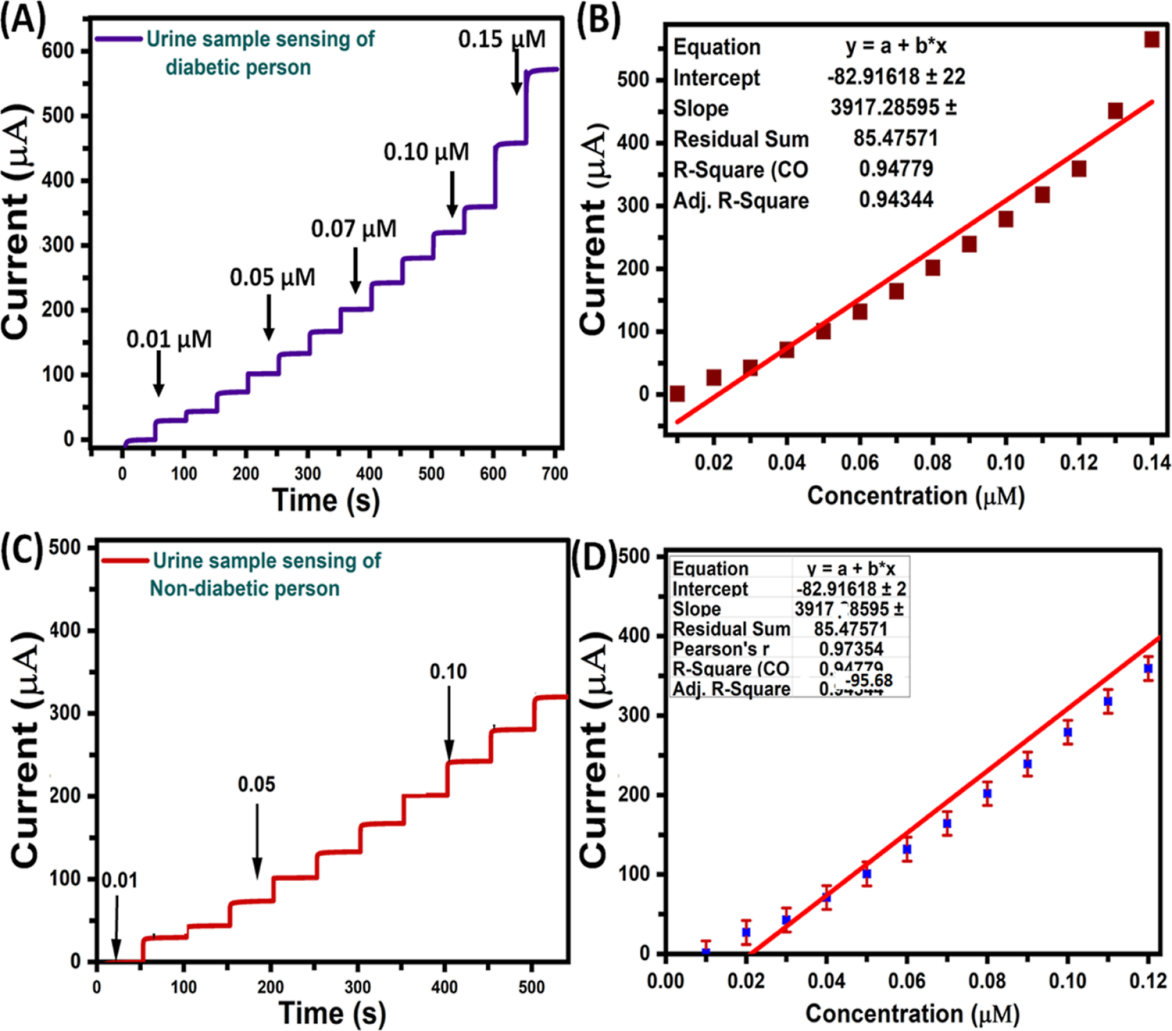
(A) Amperometry spectra and (B) different glucose concentration versus current response plot present in urine sample response of diabetic person with 0.1 M NaOH solution, (C) amperometry spectra and (D) plot illustrating the current response versus glucose concentration present in urine sample response of nondiabetic person with 0.1 M NaOH solution.

The injections of various concentrations of glucose in blood serum sample from the nondiabetic person’s and corresponding recovery results were summarized in Table 2. We have extended the detection of glucose in urine samples that obtained from diabetic person using MoS_2_-GCE nanostructures composite modified electrode by amperometry (Figure 5A).

**Table 2:** Detection of Glucose in Blood Serum Samples Collected from the Non-diabetic Person

added (mM)	found concentration (mM)	mean recovery (%)	RSD (%, *n* = 3)
0.01	0.029451	93.60	1.31106
0.05	0.069451	95.76	0.56015
0.1	0.119451	98.95	1.117511
0.15	0.169451	99.99	0.387368
0.2	0.219451	99.99	0.753969

We have also added similar concentrations of glucose (0.01 to 0.2 μM) into the diabetic patient’s urine samples; the obtained amperometric *i*–*t* curve responses were highly sensitive up on each addition, and the corresponding linearity was distorted with the *R*^2^ value of 0.947 (Figure 5B). The injections of various concentrations of glucose in urine diabetic patient’s samples and corresponding recovery results were summarized in Table 3.

**Table 3:** Detection of Glucose in Urine Samples Collected from a Diabetic Patient

added (μM)	found concentration (μM)	mean recovery (%)	RSD (%, *n* = 3)
0.01	0.029065	97.25	0.023515
0.05	0.069065	96.23	0.046671
0.1	0.119065	95.55	0.088211
0.15	0.139065	99.98	0.106858
0.2	0.159065	99.96	0.154746

On the other side, the detection of glucose was performed in urine serum samples that collected from nondiabetic person (Table 4) using MoS_2_–GCE nanostructures composite modified electrode by amperometry (Figure 5C). The concentrations of glucose (0.01 to 0.2 μM) were injected into the blood serum samples of nondiabetic person’s sample, the obtained amperometric *i*–*t* curve responses were fairly less sensitive and corresponding linearity was distorted with the *R*^2^ value of 0.947 (Figure 5D), which obviously indicate the clear difference during the detection of glucose. The observed results indicate that the MoS_2_–GCE-sensing interface showed 3.68 μA/μM, response in diabetic persons, which is better sensitivity than nondiabetic patients (2.42 μA/μM) toward the detection of glucose.

**Table 4:** Detection of Glucose in Urine Samples Collected from a Non-diabetic Person

added (μM)	found concentration (μM)	mean recovery (%)	RSD (%, *n* = 3)
0.01	0.029769713	98.23	0.0224
0.05	0.049769713	95.68	0.031706
0.1	0.079769713	94.63	0.051757
0.15	0.119769713	96.68	0.08492
0.2	0.159769713	99.88	0.149217

### 3.7 Stability, Reproducibility, and Storage Test

In this study, we carefully assessed our biosensor’s performance for glucose detection across a range of clinically relevant values. Recognizing the importance of assessing repeatability and reproducibility, we performed extensive experiments with three different glucose concentrations: 30 mg/dL, which represents the lower range; 120 mg/dL, which represents normal physiological levels; and 250 mg/dL, which corresponds to elevated glucose concentrations commonly observed under hyperglycemic conditions. We wanted to quantify the biosensor’s dependability and accuracy over the dynamic glucose concentration range using a thorough experimental design that included at least 30 repeats for each concentration (10 replicates for low, 10 for medium, and 10 for high) shown in Figure S3. The repeatability of the process was rigorously assessed through 100 iterations within a single day, and this procedure was subsequently replicated over three consecutive days using a modified electrode. The relative standard deviation of the repeatability for electrode storage was determined to be 2.25%, as illustrated in Figure S4, indicating the remarkable consistency of the proposed sensor.

## 4 Conclusions

In summary, we demonstrated the next-generation 2D-MoS_2_ nanostructured sensor for diabetes management. The fabricated MoS_2_-GCE sensing interface displayed an enhanced electro-oxidation of glucose under alkaline conditions. Notably, the MoS_2_-based electrode exhibited a linear detection range of 0.01–0.20 mM and a high sensitivity of 1194.04 μA μM^–1^ cm^–2^ with the LOD of 22.08 ng mL^–1^. This work provides a facile approach for developing the MoS_2_–GCE interface as a promising solution for flexible and disposable nonenzymatic glucose sensors. Further, the proposed glucose monitoring system had high potential for assessing glucose concentrations in various biological fluids viz. blood serum and urine of diabetic and nondiabetic persons. These findings suggest that MoS_2_–GCE interface shows considerable potential as a promising option for hyperglycemia detection and therapy. Thus, the development of simple and effective NEGS offers new prospects in the glucometer industry.

## Data Availability

All data is given in the manuscript.

## Notes

Ethical Approval: This study evaluated 2D-MoS_2_ nanostructured sensor as a next-generation glucose sensor for diabetes management using real blood serum and urine samples of diabetic and nondiabetic persons, collected from the Aster MaxCare Hospital Salalah, Oman after the approval from the Dhofar University Research Ethics and Biosafety Committee (ref. no. DU-AY-22–23-RETHC-001). Moreover, all rules and regulation of handling these real biological samples in context with Oman were followed throughout the analysis.
