# Supplementary material for: Revolutionizing Glucose Monitoring: Enzyme-Free 2D-MoS2 Nanostructures for Ultra-Sensitive Glucose Sensors with Real-Time Health-Monitoring Capabilities
Source: ACS Omega. 2024 Apr 25;9(18):20021–9. doi: 10.1021/acsomega.3c10117 (PMC11079886; doi:10.1021/acsomega.3c10117)
Supplement: Supplementary file 1 — ao3c10117_si_001.pdf [file ao3c10117_si_001.pdf]

Revolutionizing Glucose Monitoring: Enzyme-Free 2D-MoS_2 Nanostructures for Ultra-Sensitive Glucose Sensors with Real-time Health Monitoring Capabilities

Mustri Bano[a*], Gowhar A. Naikoo[a*], Fatima BaOmar[a], Jahangir A. Rather[b*], Israr U. Hassan[a], Rayees A. Sheikh[c], Palanisamy Kannan[d], Murtaza M. Tambuwala[e*]

[a]*Department of Mathematics and Sciences, College of Arts and Applied Sciences, Dhofar University, Salalah, PC 211, Oman*

[b]*Department of Chemistry, Sri Pratap College Srinagr-190001, Kashmir, India.*

[c] *Department of Chemistry, AAAM Degree College Bemina Srinagar – 190018, Kashmir, India.*

[d]*College of Biological, Chemical Sciences and Engineering, Jiaxing University Jiaxing, 314001, PR China.*

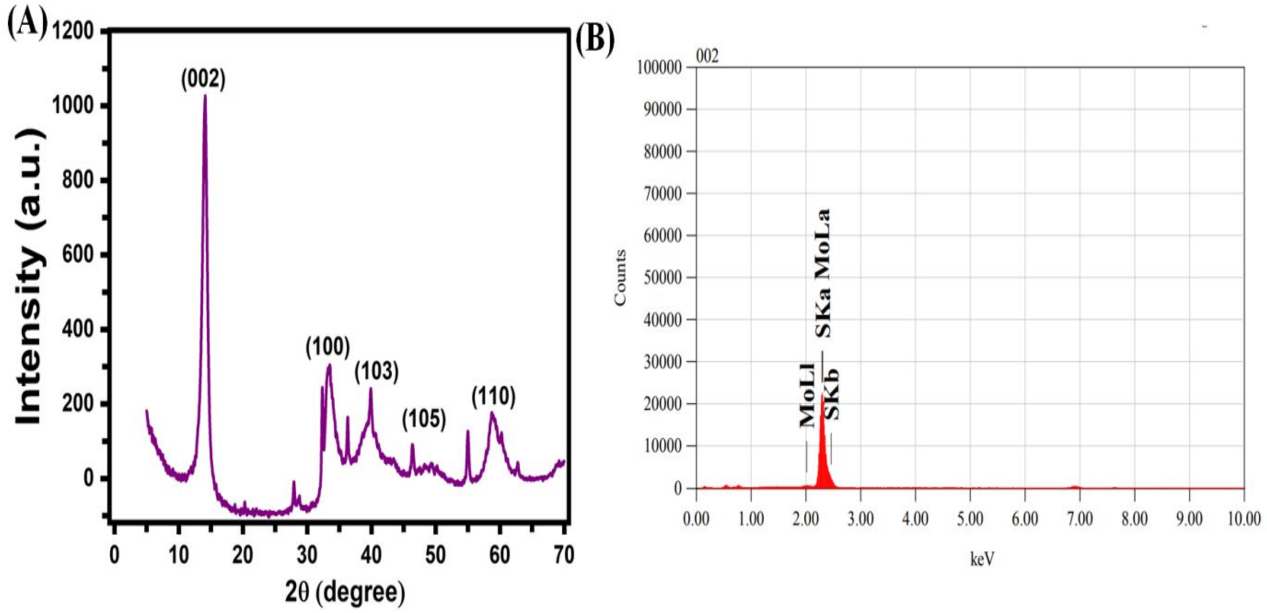

Figure S1. (A) XRD, and **(B)** EDX of 2D-MoS_2 nanostructures materials.

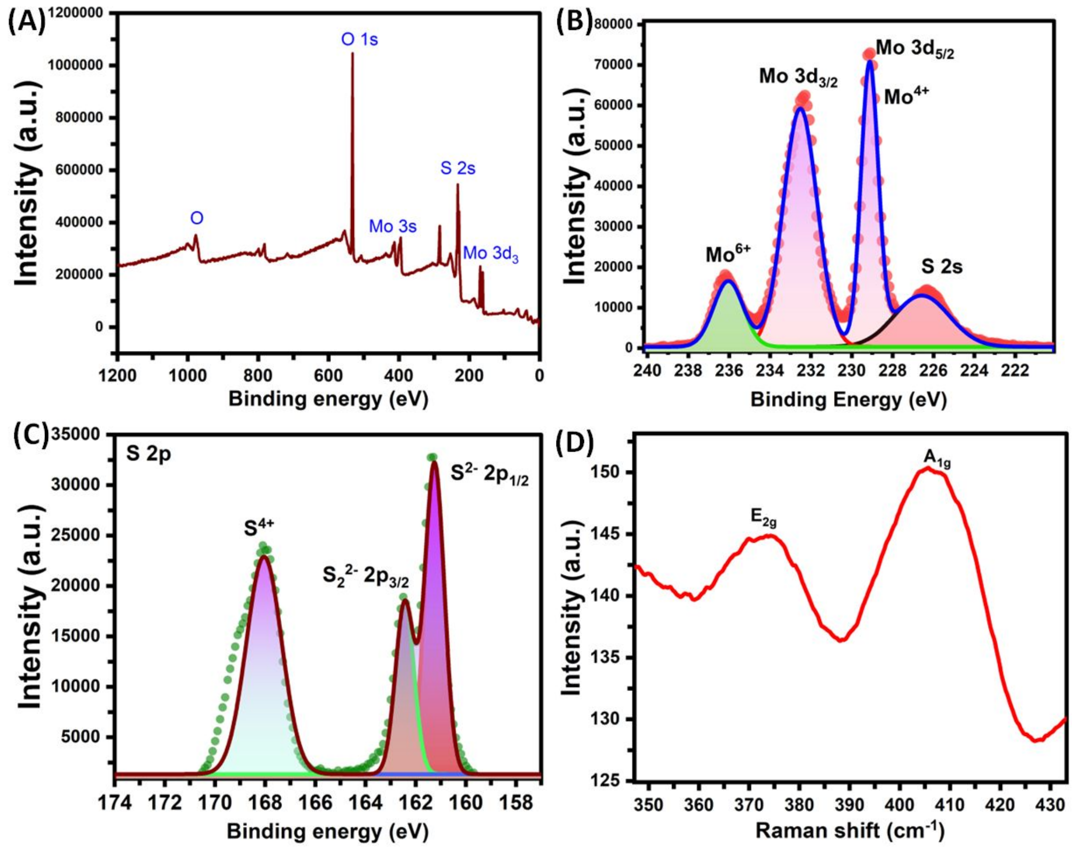

Figure S2. (A) Combined graph of 2D-MoS_2 XPS, **(B)** Mo $3d_{5/2}$, **(C)** S^{2-} $2p_{1/2}$, **(D)** Raman spectra of 2D-MoS_2 nanostructures.

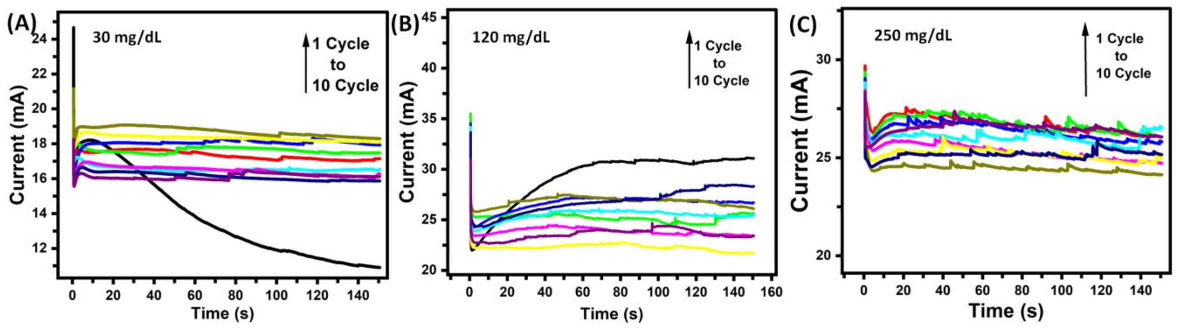

Figure S3. Glucose concentration range (A) 30 mg/dL, (B) 120 mg/dL and (C) 250 mg/dL with 0.1 M NaOH of 2D-MoS_2 nanostructures materials.

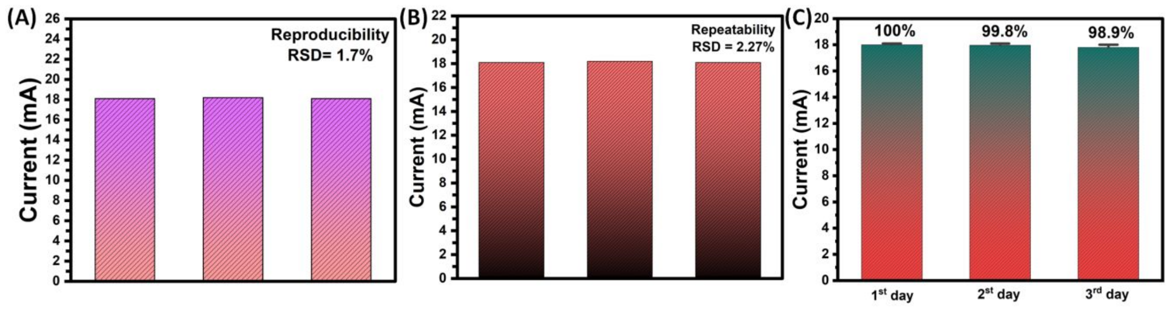

Figure S4. (A) Reproducibility, (B) Repeatability and (C) Stability and Storage test of 2D-MoS_2 nanostructures materials.
